# Circulating Tumor DNA as a Predictive Marker of Recurrence for Patients With Stage II-III Breast Cancer Treated With Neoadjuvant Therapy

**DOI:** 10.3389/fonc.2021.736769

**Published:** 2021-11-12

**Authors:** Po-Han Lin, Ming-Yang Wang, Chiao Lo, Li-Wei Tsai, Tzu-Chun Yen, Thomas Yoyan Huang, Wei-Chih Huang, Karen Yang, Chih-Kai Chen, Sheng-Chih Fan, Sung-Hsin Kuo, Chiun-Sheng Huang

**Affiliations:** ^1^ Department of Medical Genetics, National Taiwan University Hospital, Taipei, Taiwan; ^2^ Institute of Medical Genomics and Proteomics, College of Medicine, National Taiwan University, Taipei, Taiwan; ^3^ Department of Surgery, National Taiwan University Hospital, Taipei, Taiwan; ^4^ Department of Molecular Biology, Princeton University, Princeton, NJ, United States; ^5^ Department of Medical Oncology, National Taiwan University Hospital, Taipei, Taiwan; ^6^ Department of Surgery, College of Medicine, National Taiwan University, Taipei, Taiwan

**Keywords:** circulating tumor DNA, neoadjuvant therapy, breast cancer, recurrence, next-generation sequencing

## Abstract

**Background:**

Patients with stage II to III breast cancer have a high recurrence rate. The early detection of recurrent breast cancer remains a major unmet need. Circulating tumor DNA (ctDNA) has been proven to be a marker of disease progression in metastatic breast cancer. We aimed to evaluate the prognostic value of ctDNA in the setting of neoadjuvant therapy (NAT).

**Methods:**

Plasma was sampled at the initial diagnosis (defined as before NAT) and after breast surgery and neoadjuvant therapy(defined as after NAT). We extracted ctDNA from the plasma and performed deep sequencing of a target gene panel. ctDNA positivity was marked by the detection of alterations, such as mutations and copy number variations.

**Results:**

A total of 95 patients were enrolled in this study; 60 patients exhibited ctDNA positivity before NAT, and 31 patients exhibited ctDNA positivity after NAT. A pathologic complete response (pCR) was observed in 13 patients, including one ER(+)Her2(-) patient, six Her2(+) patients and six triple-negative breast cancer (TNBC) patients. Among the entire cohort, multivariate analysis showed that N3 classification and ctDNA positivity after NAT were independent risk factors that predicted recurrence (N3, hazard ratio (HR) 3.34, 95% confidence interval (CI) 1.26 – 8.87, p = 0.016; ctDNA, HR 4.29, 95% CI 2.06 – 8.92, p < 0.0001). The presence of ctDNA before NAT did not affect the rate of recurrence-free survival. For patients with Her2(+) or TNBC, patients who did not achieve pCR were associated with a trend of higher recurrence (p = 0.105). Advanced nodal status and ctDNA positivity after NAT were significant risk factors for recurrence (N2 – 3, HR 3.753, 95% CI 1.146 – 12.297, p = 0.029; ctDNA, HR 3.123, 95% CI 1.139 – 8.564, p = 0.027). Two patients who achieved pCR had ctDNA positivity after NAT; one TNBC patient had hepatic metastases six months after surgery, and one Her2(+) breast cancer patient had brain metastasis 13 months after surgery.

**Conclusions:**

This study suggested that the presence of ctDNA after NAT is a robust marker for predicting relapse in stage II to III breast cancer patients.

## Introduction

Although breast cancer prognosis has improved during the past two decades, breast cancer-related death remains a major cause of cancer-related mortality in women (1, 2). The main reason is that a significant proportion of breast cancer patients develop recurrence and distant metastases (3, 4). Once metastases occur, breast cancer is treatable but no longer curable (5).

For breast cancer patients, early detection of recurrence remains a major unmet need. In the neoadjuvant setting, pathological complete response (pCR) is a favorable prognostic marker in patients with Her2 (+) and triple-negative breast cancer (TNBC) (6). However, some patients with pCR may still experience recurrence or metastasis; on the other hand, the absence of pCR does not necessarily correlate with recurrence (6, 7). Recent studies have shown circulating tumor DNA (ctDNA), which are circulating DNA fragments that carry tumor-specific sequence alterations found in the cell-free fraction of blood, to be a promising and sensitive tool for targeted monitoring (8–12). The detection of resistance mutations using ctDNA can also occur significantly earlier than radiographic progression (13). In previous reports of metastatic cancer patients, serial quantification of ctDNA allowed for noninvasive assessment of therapeutic response and understanding of resistance mechanisms (8, 11, 14, 15). For patients with early-stage breast, lung and colon cancer, studies reported that ctDNA in the plasma can be used to detect minimal residual disease (16–18). Serial detection of ctDNA after surgery and adjuvant chemotherapy of breast cancer could identify recurrent disease earlier than clinical overt tumor presenting in the radiologic images (19, 20). However, for breast cancer patients receiving neoadjuvant therapy (NAT), the prognostic value of ctDNA before and after NAT is uncertain. It is unknown whether ctDNA or pCR has a more prognostic value for breast cancer patients, either. To determine the prognostic value of ctDNA in the context of NAT, we collected the patients’ plasma before and after NAT and used next-generation sequencing (NGS)-based deep sequencing to detect ctDNA and evaluated the impact of ctDNA on disease recurrence.

## Methods

### Patients and Sample Collection

Stage II or III breast cancer patients who received NAT were enrolled in this study. The clinical and pathologic characteristics were reviewed retrospectively from medical records. The presence of estrogen receptors (ER), progesterone receptors (PR), and Her2 were determined by immunohistochemical staining. The ER or PR status was considered negative when less than 1% of the tumor cells showed positive staining. For Her2 staining, a score of 0 or 1+ was considered negative; specimens with a score of 2+ were further tested with fluorescence *in situ* hybridization analysis. The tumor histological grade was defined using the Nottingham combined histological grading system. This study was approved by the institutional review board (IRB number: 201704009RINC).

At the initial diagnosis (defined as before NAT), a 10-mL sample of blood was collected and stored in an EDTA-containing tube. Then, all patients were treated with NAT and received breast surgery. After NAT and breast surgery (defined as after NAT), another 10 mL of blood was sampled. Within three hours of blood sampling, the plasma was extracted after centrifugation at 1000× G for 10 minutes then stored at -80°C (21). Cell-free DNA was extracted using a QIAamp Circulating Nucleic Acid Kit (Qiagen, Germantown, MD, USA) according to the manufacturer’s protocol.

### Library Preparation and Next-Generation Sequencing

The library was constructed using a QIAseq Targeted DNA Panel with a customized gene list. The customized panel was designed to amplify the coding regions of the following genes: *TP53*, *PIK3CA*, *Her2*, *GATA3*, *CDH1*, *PTEN*, *AKT1*, *ESR1*, *S100A7-9*, *ZNF703*, *B2M*, *CCND1*, *GATA3* and *c-MYC*. According to the manufacturer’s protocol, 10 ng of DNA was digested briefly into small fragments by a fragmentation enzyme at 32°C and 72°C. The DNA fragments were added to the QIAseq IL-N7 adapters, followed by target enrichment polymerase chain reaction (PCR) using the QIAGEN IL-Forward primer and the targeted DNA Panel primers. Finally, the library was amplified with universal PCR. The DNA library was then checked by using an Agilent Chip High Sensitivity DNA kit. KAPA library quantification kits were used to quantify the final concentration. The final DNA library was sequenced with the following Illumina platforms: Illumina MiSeq Reagent Kit v2, 2 x 150 bp reads or Illumina NextSeq 550 system Mid-Output Kit, 2 x 150 bp reads.

### Post-Sequencing Analysis

Previously, we have constructed an analytic pipeline of post‐NGS bioinformatics (22). First, BWA software (version 0.5.9) was used to align the raw sequencing data to the reference human genome [Feb. 2009, GRCh37/hg19; SAMtools (version 0.1.18)]. Picard (version 1.54) was used to perform the necessary data conversion, sorting, and indexing. GATK was used for variant calling with the Mutect2 and VariantFiltration parameters. Finally, ANNOVAR was used to annotate the genetic variants. Pathogenic and likely pathogenic variants were defined according to the American College of Medical Genomics and Genetics (ACMG) guidelines (23). The presence of ctDNA was determined by the presence of pathogenic and likely pathogenic variants, which are also considered tumor mutations. For variants of uncertain significance, if the prevalence of the variants in the normal population was less than 0.01 in a genomic database (1000 Genomics, ESP6500 and ExAC) and predicted to be deleterious by computer software (SIFT, PolyPhen2, and CADD), then they were classified as “highly suspected deleterious”. The above filtering analyses removes germline variants as much as possible (24); these variants are highly suspected to originate from tumors, so the detection of these variants could be considered indicative of ctDNA.

### Analysis of Copy Number Changes

Since the *Her2*, *c-Myc*, *CCND1* and *S100A* genes can be amplified in some breast cancer tumors, we decided to use copy number variations (CNV) to indicate the presence of ctDNA (25–27). Copy number variations were analyzed by OncoCNV (https://github.com/BoevaLab/ONCOCNV) according to the authors’ instructions. The baseline control consisted of the ctDNA BAM files of 14 healthy people. The ctDNA BAM files from the breast cancer patients were compared to the BAM files from the control population by using OncoCNV’s default *cghseg* segmentation algorithm (28). The sequencing region of each targeted gene was divided into several segments. When the mean of all segments of each gene was significantly different from the baseline, such as when the copy number predicted was greater than three copies or fewer than one copy from the baseline, we considered that to indicate a CNV alteration, which indicated the presence of ctDNA.

### Statistics

The chi-squared test and Fisher’s exact test were used to calculate the significance of the variance between each group. Survival was estimated by Kaplan-Meier analysis. Cox proportional hazards regression analysis was used to estimate the hazards ratios of RFS with a corresponding 95% confidence interval (CI) for various factors. All *p* values are two-sided, and *p*-values less than 0.05 were considered statistically significant.

## Results

### Evaluation of Assay Performance

First, to confirm the accuracy of the NSG-based deep sequencing, we checked whether this method could distinguish the existence of low-abundance mutants from background errors arising from the polymerase chain reaction (PCR) or sequencing process. We constructed a *TP53* mutant (NM_000546.6: c.844C>A) as a reference sample; then we utilized this *TP53* mutant with serial concentrations of 100%, 10%, 1%, and 0.1% to test whether the experimental method could detect these mutants at these concentrations (**Supplementary Methods**). The results demonstrated that the signal from the 0.1% mutant was significantly higher than background errors (**Supplementary Figure S1A**), suggesting that NGS testing accurately detected mutants present at 0.1%. In addition, the mutation level could be measured with a linear fashion (R^2^ = 0.9997, **Supplementary Figure S1B**).

Second, in deep cell-free analyses, another source of variants that makes it hard to distinguish cancer mutations is clonal hematopoiesis of indeterminate potential (CHIP) (29–31). The CHIP mutations mostly occur in the *DNMT3A*, *TET2*, *PPM1D*, *ASXL1* and *TP53* genes (29), whereas pathogenic variants of breast cancer were most prevalent in *TP53*, *PIK3CA*, *MAP3KA1*, *CDH1*, and *PTEN* (32). Variants most likely to be indistinguishable from CHIP were located in *TP53*. Twenty-two tumors from the pre-neoadjuvant core biopsy tumors were available for DNA extraction and sequencing (**Supplementary Table S1**). Among them, 6 patients had *TP53* variants, and their *TP53* variants co-existed in the ctDNA and DNA from tumor biopsies (**Supplementary Table S1** and **Supplementary Figure S2**), suggesting the *TP53* variants origin from breast cancer, not CHIP mutations.

### Patients

A total of 95 patients were enrolled in this study. The median age was 50.0 years old. Forty-one patients had ER(+) Her2(-) breast cancer, 29 patients had Her2(+) breast cancer, and 25 patients had triple-negative breast cancer (TNBC). Before NAT, tumors with T1, T2 and T3-4 size classifications were found in three, 54 and 38 patients of each population, respectively. Eighty-two patients had positive axillary lymph nodes. According to standard clinical practice, ER(+) Her2(-) breast cancer patients with large tumors were treated with NAT. Out of the 95 patients, 77 patients received anthracycline while 80 patients received taxane in their NAT regimens. All Her2(+) patients received adjuvant anti-Her2 target therapy (27 patients receiving trastuzumab, one another receiving trastuzumab/pertuzumab and the other receiving trastuzumab-DM1). After NAT, 13 patients achieved a pCR of their primary breast tumors; 82 patients did not have pCR. Among the 13 pCR patients, there was one ER(+) Her2(-), six Her2(+) and six TNBC patients. The frequency of pCR was significantly higher in patients with Her2(+) breast cancer or TNBC than ER(+)Her2(-) patients (p = 0.002). CtDNA was detected in 60 patients before NAT and 31 patients after NAT. All of the clinical and pathologic characteristics are shown in **Table 1**.

**Table 1:** Clinical and pathologic characteristics of enrolled patients stratified by immunophenotypes.

	All	ER(+) Her2(-)	ER(±) Her2(+)	TNBC
Number	95	41	29	25
Age (mean ± SD)	50.0 ± 8.8	49.2 ± 7.8	49.3 ± 8.7	52.0 ± 10.2
T classification (before NAT)				
T1	3	0	1	2
T2	54	19	16	19
T3-4	38	22	12	4
N classification (before NAT)				
N-negative	13	4	6	3
N-positive	82	37	23	22
T classification (after NAT)				
no tumor	13	1	6	6
T1	32	10	13	9
T2	29	16	6	7
T3-4	21	14	4	3
N classification (after NAT)				
N0	34	7	16	11
N1	29	9	10	10
N2	22	17	2	3
N3	10	8	1	1
Response				
pCR	13	1	6	6
absence of pCR	82	40	23	19
NAT regimen				
Anthracycline	77	33	24	20
Taxane	80	29	29	22
Trastuzumab/pertuzumab	29	0	29	0
Presence of ctDNA				
before NAT	60	33	15	12
after NAT	31	11	10	10
Adjuvant chemotherapy	30	18	3	9
anthracycline	15	8	3	4
taxane	15	10	0	5
Adjuvant anti-Her2 target therapy*	29	0	29	0

anti-Her2 target therapy*: 27 patients receiving trastuzumab, one another receiving trastuzumab/pertuzumab and the other receiving trastuzumab-DM1.

### Genetic Alterations in Tumor ctDNA

**Figure 1:**
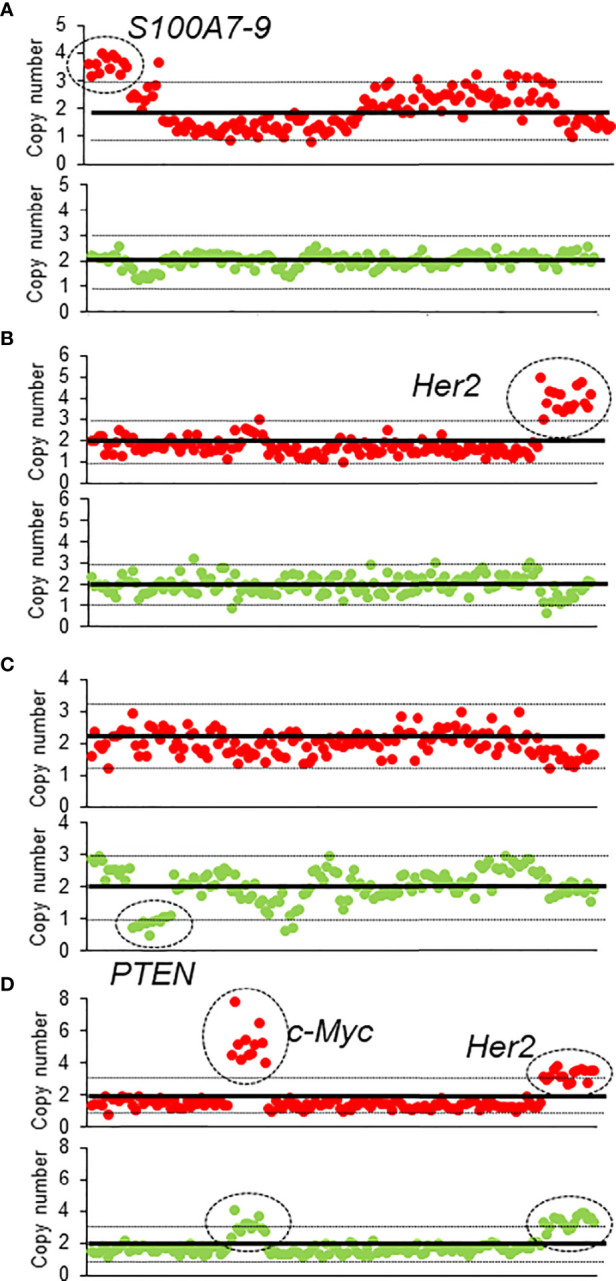
The CNV of four patients before and after NAT **(A–D)**. The red dots represent the CNV before NAT, and green dots represent the CNV after NAT.

Among the 95 patients, 19 patients were found to have ctDNA before and after NAT; 41 patients had ctDNA only before NAT, 12 patients had ctDNA only after NAT, and 23 patients had ctDNA neither before nor after NAT (**Supplementary Table S2**). The most common genetic variants were in the *TP53* (n = 28), followed by *PIK3CA* (n = 16), *CDH1* (n = 15), and *Her2* (n = 7) genes. Eighteen patients had altered CNVs in their ctDNA, including of *AKT1*, *CCND1*, *CDH1*, *c-MYC*, *Her2*, *PIK3CA*, *S100A*, and *ZNF703*, either before or after NAT (**Supplementary Table S2** and **Figure 1**). Before NAT, Patient #73 (**Figure 1A**) and Patient #24 (**Figure 1B**) exhibited copy number gains of the *S100A* and *Her*2 genes in ctDNA, respectively; after NAT, the copy numbers of these genes in ctDNA returned to normal levels. Patient #3 (**Figure 1C**) had a new copy loss of the *PTEN* gene after NAT. We observed gains of *Her2* and *c-MYC* in patient #27 (**Figure 1D**) before NAT that were only partially resolved after NAT.

### Association Between ctDNA and Clinical Characteristics

Patients who had ctDNA before NAT tended to have a larger tumor size than those who did not have ctDNA before NAT (mean 5.0 cm *vs.* 4.3 cm, p = 0.104). However, the presence of ctDNA after NAT did not correlate with the tumor size or LN numbers after NAT. Although the difference was not statistically significant, patients with pCR had a lower detection of ctDNA after NAT than patients with no pCR (patients with pCR *vs.* absence of pCR: 15.4% *vs.* 35.4%, p = 0.132). Additionally, the presence of ctDNA was not correlated with the immunophenotype of breast cancer.

### Impact of Clinical Factors and ctDNA on RFS

**Figure 2:**
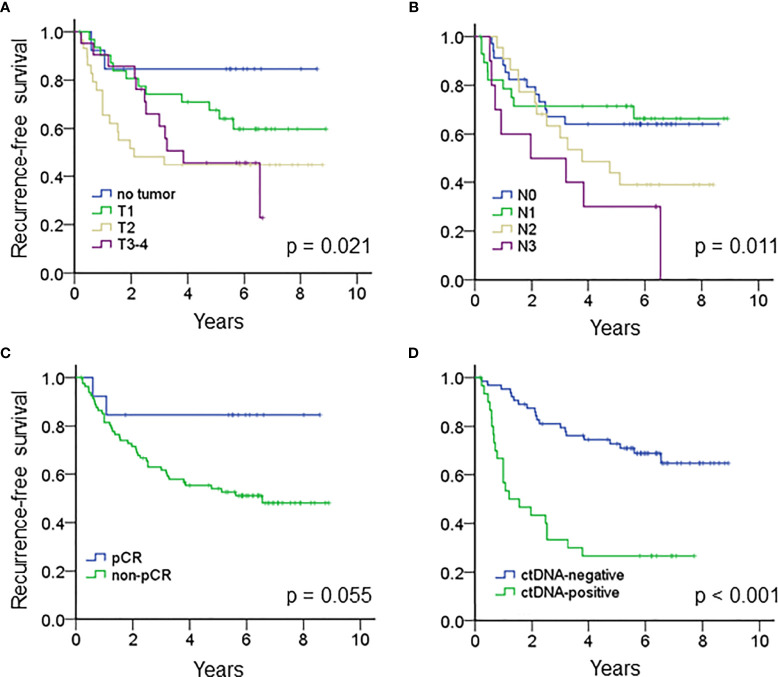
Kaplan-Meier analysis estimated the recurrence-free survival of the entire cohort according to **(A)** the tumor size after NAT (p = 0.021), **(B)** N classification after NAT (p = 0.011), **(C)** pCR (p = 0.055) and **(D)** ctDNA after NAT (p < 0.001).

The median follow-up time of the entire cohort was 5.1 years, and the 5-year recurrence-free survival (RFS) was 58% (95% CI 48.0 – 68.0%). For clinical factors, Kaplan-Meier analysis showed that the residual tumor size after NAT and N classification after NAT were prognostic factors for RFS; patients who achieved pCR tended to have a better RFS than patients who did not achieve pCR (**Figures 2A–C** and **Table 2**). On the other hand, patients with ctDNA after NAT had a significantly inferior RFS (p < 0.001, **Figure 2D**). Other factors, such as age, ctDNA detection before NAT, immunophenotype, initial tumor size before NAT and N classification before NAT and adjuvant chemotherapy did not influence RFS. RFS was similar between patients with and without *TP53*, *PIK3CA* and *CDH1* mutations (**Table 2** and **Supplementary Table S3**).

**Table 2:** Univariate and multivariate analysis of recurrence-free survival of the entire cohort.

variables	univariate				multivariate			
	HR	lower	upper	P value	HR	lower	upper	P value
Age (>50 *vs.* <50)	0.962	00.525	1.763	.899				
T classification (before NAT)								
T1-2	1							
T3-4	1.026	.553	1.903	0.936				
N classification (before NAT)								
N-negative	1							
N-positive	2.266	0.700	7.336	0.172				
T classification (after NAT)								
no tumor	1				1			
T1	2.536	0.568	11.333	0.223	1.963	0.333	11.575	0.456
T2	4.842	1.112	21.083	0.036	2.435	0.450	13.186	0.302
T3-4	4.158	0.929	18.604	0.062	2.338	0.488	11.202	0.288
N classification (after NAT)								
N0	1				1			
N1	0.953	0.401	2.263	0.914	1.378	.526	3.606	0.514
N2	1.750	0.798	3.838	0.163	1.418	.611	3.293	0.416
N3	3.055	1.246	7.487	0.015	3.352	1.267	8.870	0.015
Response								
pCR	1				1			
absence of pCR	3.656	0.883	15.134	0.074	2.230	0.468	10.623	0.314
Immunophenotype								
ER/PR(+)Her2(-)	1							
ER/PR(+)Her2(+)	0.611	0.284	1.314	0.207				
TNBC	1.294	0.639	2.622	0.474				
ctDNA								
before NAT^*^	0.700	0.378	1.298	0.257				
after NAT^*^	3.894	2.113	7.177	<0.001	4.135	2.014	8.491	<0.001
Adjuvant chemotherapy								
No	1							
Yes	1.141	0.601	2.169	0.686				
Genes								
TP53^#^	1.156	0.609	2.197	0.657				
CDH1^#^	0.669	0.263	1.704	0.399				
PIK3CA^#^	1.313	0.607	2.837	0.489				

^*^The presence of ctDNA vs. nonpresence of ctDNA; ^#^gene mutation vs. nonmutation.

We then analyzed the clinical and pathologic characteristics of patients with and without ctDNA after NAT, and no difference was found between the two patient groups (**Supplementary Table S4**). After incorporating the residual tumor size, N classification after NAT, pCR and ctDNA after NAT, multivariate analysis showed that an N3 classification and ctDNA positivity after NAT were independent risk factors that predicted tumor recurrence (N3, hazard ratio (HR) 3.352, 95% CI 1.267 – 8.870, p = 0.015; ctDNA, HR 4.135, 95% CI 2.014 – 8.491, p < 0.0001). Other factors did not significantly impact RFS (**Table 2**).

Next, we analyzed the 72 patients with detected ctDNA, either before or after NAT. Patients with ctDNA positivity after NAT had a significantly inferior RFS compared to those without detectable ctDNA (**Supplementary Figure S3**, p<0.001). After adjusting for tumor size (after NAT), N classification (after NAT) and pCR, multivariate analysis with the Cox model revealed that ctDNA positivity after NAT was the most significant risk factor that predicted tumor recurrence (HR 8.02, 95% CI 3.24 – 19.86, p < 0.0001) (**Supplementary Table S5**).

### The Impact of ctDNA on Disease Recurrence in Different Immunophenotypes of Breast Cancer

**Figure 3:**
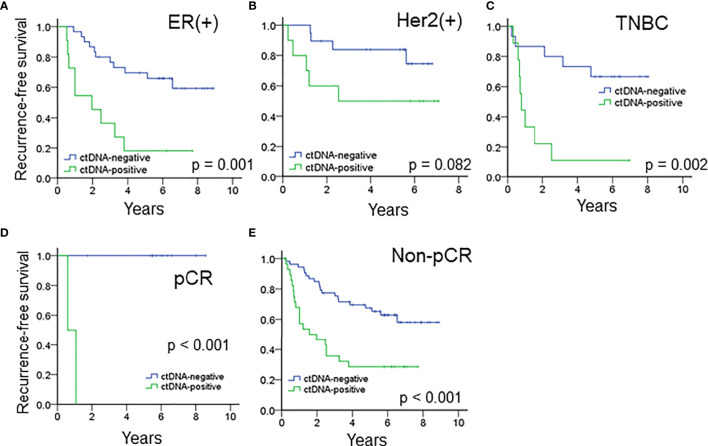
The prognostic impact of ctDNA after NAT in patients with **(A)** ER(+) breast cancer, **(B)** Her2(+) breast cancer and **(C)** TNBC. ctDNA after NAT predicted RFS in **(D)** pCR and **(E)** patients who did not achieve pCR.

The median RFS of all the patients with ctDNA positivity after NAT was 1.19 years. When stratified by the immunophenotypes, ctDNA positivity after NAT was associated with a significantly inferior RFS for ER(+) breast cancer or TNBC patients and a trend of higher recurrence rates for patients with the Her2 subtype (**Figures 3A–C**). The median RFS of ER(+) breast cancer, Her2 (+) breast cancer and TNBC patients with ctDNA positivity after NAT were 0.90, 2.52 and 0.74 years, respectively.

### The Impact of ctDNA on Disease Recurrence in Patients With and Without a pCR

For the entire cohort, the presence of ctDNA after NAT was a significant risk factor associated with recurrence in both patients who achieved and did not achieve pCR (**Figures 3D, E**, all p < 0.001). Because pCR was previously reported as a surrogate marker for survival in patients with Her2(+) and TNBC (6), we analyzed these patient subgroups. Between the two patient populations, pCR was related to a trend of improved survival compared to absence of pCR (HR 3.328, 95% CI 0.777 – 14.243, p = 0.105, **Supplementary Table S6**). Multivariate analysis showed that advanced nodal status and ctDNA after NAT were independently correlated with high risk (N2-3, HR 3.753, 95% CI 1.146–12.297, p = 0.029; ctDNA, HR 3.123, 95% CI. 1.139 – 8.564, p = 0.027), and pCR status did show a not significant correlation with recurrence (**Table 3**). A potential reason for this phenomenon is that pCR only represents the therapeutic efficacy of local breast tumor and the ctDNA may indicate that an occult lesion is present that is not effectively treated with NAT. In our study, 13 patients achieved pCR after NAT, and among those patients, two exhibited ctDNA positivity after NAT. One TNBC patient (case #50) received neoadjuvant docetaxel/epirubicin (four cycles) and achieved pCR for her primary breast and axillary tumors. However, she had hepatic metastases at 6 months after mastectomy (**Supplementary Figure S4**). The other patient (case #5) had Her2-positive breast cancer and received neoadjuvant docetaxel/trastuzumab (four cycles) and epirubicin/cyclophosphamide (four cycles). The pathology showed no residual tumors. Trastuzumab was continuously maintained for one year. At the end of trastuzumab treatment (13 months after mastectomy), a cerebellar metastasis was found. The other 11 patients who achieved a pCR did not have ctDNA after NAT nor did they experience recurrence or metastasis.

**Table 3:** Multivariate analysis of recurrence-free survival in patients with Her2(+) breast cancer and TNBC.

Variables	HR	lower	upper	P value
T classification (after NAT)				
no tumor	1			
T1	0.909	0.167	4.952	0.912
T2	2.461	0.435	13.917	0.308
T3-4	4.082	0.756	22.038	0.102
N classification (after NAT)				
N0	1			
N1	1.845	.633	5.378	0.262
N2-3	3.753	1.146	12.297	0.029
Response				
pCR	1			
absence of pCR	4.082	0.756	22.038	0.102
Adjuvant chemotherapy				
No	1			
Yes	1.137	0.419	3.084	0.801
ctDNA after NAT				
undetected	1			
detected	3.123	1.139	8.564	0.027

## Discussion

Our data suggested that the presence of ctDNA after NAT is a prognostic factor that predicts breast cancer recurrence after mastectomy. Traditionally, the therapeutic response to NAT was considered a marker for predicting prognosis (6). In our study, multivariate analysis showed a greater predictive value for ctDNA than the response of the primary breast tumor to NAT treatment. Therefore, ctDNA seems more representative of the therapeutic efficacy of primary and potential micrometastatic tumors treated with NAT.

During the median 5.1-year follow-up, the overall positive predictive value of ctDNA positivity after NAT for disease relapse was 70.9%, which was higher than the predictive value of 48.8% for relapse in patients who did not achieve pCR. After stratifying patients into pCR and absence of pCR, ctDNA positivity after NAT remained a significant risk factor for RFS among the two patient groups (**Figures 3D, E**). Although patients who did not achieve pCR usually had a significantly inferior RFS than pCR patients, ctDNA negativity after NAT in patients who did not achieve pCR was associated with a better RFS (**Figure 3E**), compatible with previous findings that ctDNA clearance associated with the improved survival in patients who did not achieve pCR (33). In contrast, pCR after NAT was a surrogate marker for predicting disease-free Her2(+) and TNBC patients. However, in our cohort, two patients (one Her2(+) and one TNBC) who achieved a pCR and exhibited ctDNA positivity after NAT developed distal metastasis at six months and one year, respectively. A possible reason is that the pCR was assessed using only primary breast tumor detection without evaluating systemic micrometastatic tumor cells. The patient who had Her2-positive breast cancer and achieved a pCR after NAT developed brain metastasis after trastuzumab maintenance therapy. This was compatible with previous report that trastuzumab was difficult to penetrate the blood-brain barrier to treat brain micrometastatic tumor cells (34). However, ctDNA positivity suggested that ctDNA could cross the blood–brain barrier to be detected in the plasma (35). Thus, ctDNA is more suitable than pCR for representing the overall disease state and could be a robust marker for predicting the survival rate.

Although patients with ctDNA positivity after NAT had inferior RFS, the length of RFS varied among patients with different immunophenotypes. Among patients with ctDNA positivity after NAT, patients with Her2- positive breast cancer had a significantly longer RFS than patients with TNBC and luminal breast cancers. The maintenance of anti-Her2 antibody therapy and the potential long-term preservation of antibody-dependent cellular cytotoxicity may explain the risk attenuation and delayed relapse of Her2-positive breast cancer patients (36). In this study, some patients received adjuvant chemotherapy according to physician decision. However, adjuvant chemotherapy did not influence the RFS in the overall cohort (**Table 2**) or in each subtype of breast cancer (**Supplementary Table S3**). For patients with detected ctDNA after NAT, all twelve Her2-positive breast cancer patients received postmastectomy adjuvant anti-Her2 therapy; one received trastuzumab emtansine, another received trastuzumab plus pertuzumab, and the remaining patients received trastuzumab for one year. For the eight TNBC patients, only one received adjuvant chemotherapy. Out of the eleven patients with ER(+) breast cancer, six received adjuvant chemotherapy, and all of them received hormone therapy. Notably, the median RFS of TNBC and ER(+) breast cancer patients was less than one year. This result might suggest that current standard chemotherapy and hormone therapy treatments were not effective for these patients. CtDNA has the potential to identify actionable genetic variants that provide sensitivity or resistance mechanisms for chemotherapy and/or targeted therapy (37); this information can be used to guide personalized therapy in the future (38). Alternative adjuvant therapy options can be explored for these patients.

**Figure 4:**
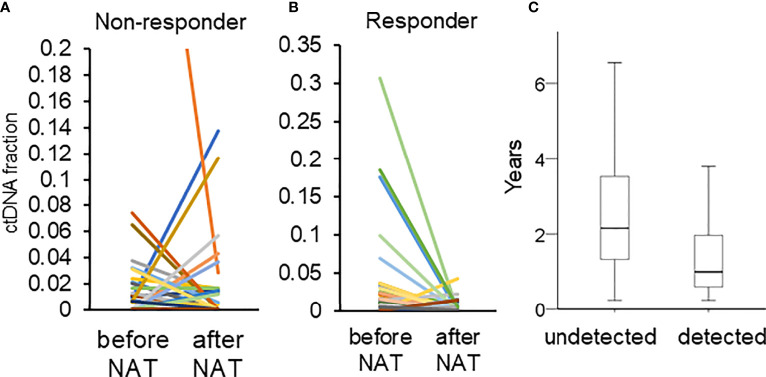
**(A, B)** Changes in the fraction of ctDNA in patients who did and did not respond. The different color represented different mutations. **(C)** The duration of RFS in patients with (detected) and without (undetected) ctDNA after NAT.

The concordance between pCR and the clearance of ctDNA was moderate. The ctDNA concentration usually decreases after NAT (17, 39). In a previous report, the decrease in ctDNA levels in patients who achieved a pCR was greater than that in those who did not achieve a pCR (39). Similarly, our data revealed that a lower proportion of patients who achieved a pCR exhibited ctDNA positivity after NAT than that in patients who did not achieve pCR (pCR *vs.* absence of pCR: 15.4% *vs.* 35.4%, p = 0.132). Among the 72 patients with ctDNA positivity (before and after NAT), 81.0% of responders had a decrease in ctDNA (defined as a tumor size reduction of more than 30% of the original size) (40), whereas 58.9% of nonresponders had a decrease in ctDNA concentrations (Pearson’s chi-squared, p = 0.088, **Figures 4A, B**).

One limitation to this study is the possibility that some ctDNA mutations may have originated from CHIP mutations (41). Although we observed a good concordance of genetic variants between ctDNA and available pre-neoadjuvant biopsy tumors, the possibility that some ctDNA mutations originated from CHIP mutations could not be ruled out because we did not have all of the biopsy tumors for sequencing. To reduce the possibility of detecting CHIP mutations, first, we designed a sequencing panel by selecting genes that are often mutated in breast cancer, not in hematologic cells (32). This strategy decreases the possibility of mixing the CHIP mutations into breast cancer mutations. Second, we only considered pathogenic/likely pathogenic or highly-suspicious deleterious variants as proof of ctDNA positivity. These variants may have biological implications for breast cancer. For example, PIK3CA H1047R is a driver mutation in breast cancer (42), suggesting that it could be a ctDNA specific to breast cancer. Third, we not only analyzed the genetic variants but also the CNV. The amplification of *Her2*, *S100A* and *CCND1* have biological significance in breast cancer pathology (25, 43), and amplification of *c-MYC* is related to high-grade malignancy (44). These CNVs are considered to be derived from breast cancer. Thus, we can reduce the possibility to contaminate CHIP mutations in the ctDNA.

The second limitation was that we only examined ctDNA before and after NAT and did not perform longitudinal monitoring; as a result, we were not able to detect late recurrence. In our cohort, 42 patients had disease recurrence. Out of those 42 patients, 22 exhibited ctDNA positivity after NAT. The 22 patients with ctDNA positivity had a significantly shorter time to recurrence than those with ctDNA negativity (with ctDNA *vs.* without ctDNA: 1.31 *vs.* 2.64 years, p = 0.004, **Figure 4C**). A single time point sample of ctDNA after NAT was a significant predictor of only early recurrence. Longitudinally tracking ctDNA may improve the predictive value for both early and late recurrence (19, 20, 39).

## Conclusions

We showed that ctDNA detection after NAT has great clinical utility potential as a prognostic marker in patients with breast cancer. CtDNA detection can identify and define a subset of high-risk patients. The next step is to determine the type of adjuvant therapy strategies that can effectively reduce recurrence. Since actionable genetic variants can be detected by ctDNA, further prospective trials should focus on incorporating ctDNA detection and exploring how to guide patient treatment, which could maximize the utility of ctDNA detection.

## Data Availability Statement

The datasets presented in this study can be found in online repositories. The names of the repository/repositories and accession number(s) can be found in the article/**Supplementary Material**.

## Ethics Statement

Ethical approval was obtained from the ethical committees of National Taiwan University Hospital (IRB number: 201704009RINC). The patients/participants provided their written informed consent to participate in this study.

## Author Contributions

C-SH had full access to all the data in the study and takes responsibility for the integrity of the data and the accuracy of the data analysis. Study concept and design: P-HL and C-SH. Patient collection: P-HL, M-YW, LW-T, CL, S-HK, and C-SH. Performing experiments and bioinformatics: T-CY, TH, C-KC, KY, W-CH, and S-CF. Acquisition, analysis, or interpretation of data: all authors. Drafting of the manuscript: P-HL and C-SH. Critical revision of the manuscript for important intellectual content: all authors.

## Funding

This work was supported, in part, by research grants from the National Taiwan University Hospital (NTUH. 107-004068 and 108-004128, to P-HL) and the Ministry of Science and Technology (MOST 104-2314-B-002-106-MY3 to C-SH, and MOST 109WFA0111726 to P-HL). 

## Conflict of Interest

The authors declare that the research was conducted in the absence of any commercial or financial relationships that could be construed as a potential conflict of interest.

## Publisher’s Note

All claims expressed in this article are solely those of the authors and do not necessarily represent those of their affiliated organizations, or those of the publisher, the editors and the reviewers. Any product that may be evaluated in this article, or claim that may be made by its manufacturer, is not guaranteed or endorsed by the publisher.

## Abbreviations

ctDNA, Circulating tumor DNA; CHIP, Clonal hematopoiesis of indeterminate potential; ER, Estrogen receptor; Her2, Human epidermal growth factor receptor; NAT, Neoadjuvant therapy; pCR, Pathologic complete response; RFS, Recurrence-free survival; TNBC, Triple-negative breast cancer.
