# Supplementary material for: Circulating Tumor DNA as a Predictive Marker of Recurrence for Patients With Stage II-III Breast Cancer Treated With Neoadjuvant Therapy
Source: Front Oncol. 2021 Nov 12;11:736769. doi: 10.3389/fonc.2021.736769 (PMC8632818; doi:10.3389/fonc.2021.736769)
Supplement: Supplementary file 1 [file DataSheet_1.pdf]

Supplementary methods

Evaluation of assay performance in reference samples

To confirm the accuracy of the NSG-based deep sequencing method, we constructed a mutant: a *TP53* mutant (NM_000546.6: c.844C>A) in a pcDNA3.1 vector. Then, we mixed the mutant and wild type to at ratios of 100%, 10%, 1%, and 0.1% by using a 1: 10 serial dilution method. The above samples with different mutant concentrations were used to test whether the experimental method could detect these mutants.

First, we needed to distinguish the true existence of low-abundance mutants from background errors due to polymerase chain reaction (PCR) or deep sequencing. We calculated the mean and standard error (SD) of the background errors of each DNA region from raw FASTQ data with a quality score of 30. The mean background error per base was 9.2×10^{-4}, and the SD was 5.09×10^{-5}. The upper limit of the 99% confidence interval for background errors was 2.23×10^{-3} (Supplementary Figure S1A). The measured level of the 0.1% mutant was 3.17×10^{-3},which is significantly higher than the range of background errors. This result suggests that our NGS testing procedures can accurately detect 0.1% mutants. Second, we observed that the mutation level could be measured in a linear manner. In reference samples, we achieved a mutation-level sensitivity of 99.50%, 11.76%, 1.35% and 0.32% for 100%, 10%, 1%, and 0.1% mutants, respectively, consistent with the decreasing number of mutant molecules (Supplementary Figure S1B).

Supplementary Table S1. Sequencing results of biopsy tumors, ctDNA before and after neoadjuvant therapy

patient	Genetic alteration in tumor	Genetic alteration in ctDNA before NAT	Genetic alteration in ctDNA after NAT
#2	undetected	undetected	undetected
#5	Her2 gain	Her2 gain	PTEN:NM_001304717:exon8:c.1314delA:p.K440Rfs*8
#6	Her2 gain; PIK3CA:NM_006218:exon10:c.1637A>G,p.E546R	Her2 gain; PIK3CA:NM_006218:exon10:c.1637A>G, p.E546R	undetected
#8	TP53:NM_000546:c.916C>T:p.R306*	TP53:NM_000546:c.916C>T:p.R306*	TP53:NM_000546:c.916C>T:p.R306*
#9	PTEN loss	undetected	PTEN loss, CDH1 loss
#13	S100A gain	S100A gain	
#15	undetected	undetected	PIK3CA:NM_006218:exon10:c.1634A>C,p.E545A
#19	PTEN loss, PIK3CA loss	PTEN loss, PIK3CA loss	PTEN loss, PIK3CA loss
#27	TP53:NM_000546: c.701A>G, p.Y234C; Her2 and c-MYC gain	TP53:NM_000546: c.701A>G, p.Y234C; Her2 and c-MYC gain	TP53:NM_000546: c.701A>G, p.Tyr234Cys; Her2 and c-MYC gain
#29	ZNF703 and CCND1 gain	ZNF703 and CCND1 gain	ZNF703 and CCND1 gain
#30	PIK3CA and TP53 gain	PIK3CA and TP53 gain	undetected
#47	Her2 gain; TP53:NM_000546:exon7:c.G733A:p.G245S	Her2 gain; TP53:NM_000546:exon7:c.G733A:p.G245S	undetected
#56	undetected	undetected	undetected
#57	undetected	undetected	undetected
#58	undetected	undetected	undetected
#63	S100A gain; PIK3CA: NM_006218:exon21: c.1633G>A, p.Glu545Lys	S100A gain; PIK3CA: NM_006218:exon21: c.1633G>A, p.Glu545Lys	undetected
#68	undetected	undetected	undetected
#74	Her2 gain	Her2 gain	undetected
#83	PIK3CA:NM_006218:exon21:c.3140A>G, p.H1047R; TP53:NM_000546: c.589G>A, p.V197M	PIK3CA:NM_006218:exon21:c.3140A>G, p.H1047R; TP53:NM_000546: c.589G>A, p.V197M	undetected
#87	undetected	undetected	undetected
#91	TP53:NM_000546: c.724T>C, p.C242R	TP53:NM_000546: c.724T>C, p.C242R	TP53:NM_000546: c.724T>C, p.C242R
#92	undetected	undetected	undetected

NAT: neoadjuvant therapy

Supplementary Table S2. List of the genetic alterations and their allelic frequency before and after neoadjuvant therapy

Patient	Before NAT	Genetic alteration	Allelic frequency	After NAT	Genetic alteration	Allelic frequency
#1	detected	AKT1 gain; PIK3CA:NM_006218:exon21:c.T2966C:p.L989P	0.00785	detected	AKT1 gain	
#2	undetected			undetected		
#3	undetected			detected	new PIK3CA and PTEN_loss	
#4	detected	TP53:NM_000546:exon6:c.G638A:p.R213Q	0.00949	undetected		
#5	detected	Her2 gain		detected	PTEN:NM_001304717:exon8:c.1314delA:p.K440Rfs*8	0.01165
#6	detected	Her2 gain; PIK3CA:NM_006218:exon10:c.1637A>G,p.E546R	0.17650	undetected		
#7	detected	TP53:NM_000546:exon7:c.A740G:p.N247S	0.02588	undetected		
#8	detected	TP53:NM_000546:c.916C>T:p.R306*	0.60552	detected	TP53:NM_000546:c.916C>T:p.R306*	0.13730
#9	undetected			detected	PTEN loss, CDH1 loss	
#10	detected	TP53:NM_000546:exon9:c.A978C:p.E326D	0.03797	undetected		
#11	detected	ERBB2:NM_004448:exon18:c.A2101C:p.T701P	0.02413	detected	ERBB2:NM_004448:exon18:c.A2101C:p.T701P	0.00934
#12	undetected			undetected		
#13	detected	S100A gain		detected		
#14	detected	MYC:NM_002467:exon2:c.G121A:p.D41N	0.02169	detected	MYC:NM_002467:exon2:c.G121A:p.D41N	0.01334

#15	undetected			detected	PIK3CA:NM_006218:exon10:c.1634A>C,p.E545A	0.01660
#16	undetected			undetected		
#17	undetected			undetected		
#18	detected	CDH1:NM_004360:exon12:c.C1792T:p.R598X	0.03341	undetected		
#19	detected	PTEN loss, PIK3CA loss		detected	PTEN loss, PIK3CA loss	
#20	undetected			undetected		
#21	undetected			undetected		
#22	detected	PTEN:NM_001304717:exon8:c.A1186T:p.K396X	0.02128	undetected		
#23	detected	TP53:NM_000546:exon5:c.A397G:p.M133V	0.00998	undetected		
#24	detected	Her2 gain		undetected		
#25	detected	TP53:NM_000546:exon8:c.A850G:p.T284A	0.01094	undetected		
#26	detected	TP53:NM_000546:exon6:c.C574T:p.Q192X	0.02077	undetected		
#27	detected	TP53:NM_000546: c.701A>G, p.Y234C; Her2 and c-MYC gain	0.064844	detected	TP53:NM_000546: c.701A>G, p.Tyr234Cys; Her2 and c-MYC gain	0.014906832
#28	undetected			undetected		
#29	detected	ZNF703 and CCND1 gain		detected	ZNF703 and CCND1 gain	
#30	detected	PIK3CA and TP53 gain		undetected		
#31	detected	CDH1:NM_004360:exon7:c.G841A:p.V281M	0.00793	undetected		
#32	detected	GATA3:NM_001002295:exon6:c.A1334G:p.X445W	0.01605	undetected		
#33	detected	TP53:NM_000546:exon9:c.920-2A>C	0.02182	undetected		

#34	undetected			undetected		
#35	detected	Her2 gain; B2M:NM_004048:exon1:c.T2C:p.M1T	0.03652	undetected		
#36	detected	CDH1:NM_004360:exon7:c.G856A:p.A286T	0.01074	undetected		
#37	detected	B2M:NM_004048:exon2:c.G286A:p.D96N	0.01196	undetected		
#38	undetected			undetected		
#39	undetected			undetected		
#40	detected	TP53:NM_000546:exon10:c.A1084G:p.S362G	0.00547	detected	TP53:NM_000546:exon10:c.A1084G:p.S362G	0.00757
#41	detected	ESR1 gain; TP53:NM_000546:exon5:c.455dupC:p.P153Afs	0.01277	undetected		
#42	detected	CDH1:NM_004360:exon3:c.T170C:p.F57S	0.00534	undetected		
#43	detected	TP53:NM_000546:exon8:c.G856A:p.E286K	0.00337	detected	TP53:NM_000546:exon8:c.G856A:p.E286K	0.04359
#44	detected	CDH1:NM_004360:exon7:c.A1007G:p.E336G	0.03669	undetected		
#45	detected	TP53:NM_000546: c.818G>A, p.A273H	0.069346	undetected		
#46	detected	PTEN loss; CDH1:NM_004360:exon16:c.A2448G:p.K816K	0.03246	detected	more PTEN loss; CDH1:NM_004360:exon16:c.A2448G:p.K816K	0.00349
#47	detected	Her2 gain; TP53:NM_000546:exon7:c.G733A:p.G245S	0.30619	undetected		0.00024
#48	detected	TP53:NM_000546.5:c.455dupC, p.P153Afs; PIK3CA: NM_006218: c.3026G>A, p.Gly1009Glu	0.01635; 0.60561	undetected		

#49	detected	PIK3CA:NM_006218:exon2:c.G113A:p.R38H	0.00476	undetected		
#50	undetected			detected	ERBB2:NM_004448:exon19:c.G2224A:p.D742N	0.00598
#51	detected	TP53:NM_000546:exon6:c.A641G:p.H214R		undetected		0.00000
#52	undetected			detected	TP53:NM_000546:exon5:c.C472T:p.R158C	0.01634
#53	detected	TP53:NM_000546:exon6:c.G661A:p.E221K	0.07450	undetected		
#54	detected	CDH1:NM_004360:exon16:c.A2512G:p.S838G		undetected		
#55	undetected			undetected		
#56	undetected			undetected		
#57	undetected			undetected		
#58	undetected			undetected		
#59	detected	TP53:NM_000546:exon5:c.G469A:p.V157I	0.02433	undetected		0.00000
#60	detected	ESR1:NM_000125:exon8:c.C1754T:p.T585M	0.01395	undetected		
#61	detected	CDH1:NM_004360:exon5:c.C655T:p.P219S	0.00680	undetected		
#62	undetected			undetected		
#63	detected	S100A gain; PIK3CA: NM_006218:exon21: c.1633G>A, p.Glu545Lys	0.01547	undetected		
#64	detected	ERBB2:NM_004448:exon19:c.A2297G:p.E766G	0.00229	detected	ERBB2:NM_004448:exon19:c.A2297G:p.E766G	0.02209
#65	undetected			detected	PIK3CA:NM_006218:exon5:c.G1030A:p.V344M	0.11671
#66	detected	PIK3CA:NM_006218:exon10: c.1634A>C, p.E545A		detected	PIK3CA:NM_006218:exon10: c.1634A>C, p.E545A	0.04240

#67	undetected			detected	TP53:NM_000546:exon5:c.C466T:p.R156C	0.01496
#68	undetected			undetected		
#69	detected	GATA3:NM_001002295:exon5:c.T1031C:p.L344P	0.01467	undetected		
#70	detected	TP53:NM_000546:exon5:c.G438A:p.W146X	0.00606	detected	TP53:NM_000546:exon5:c.G438A:p.W146X	0.01147
#71	detected	PIK3CA:NM_006218:exon21:c.A3062G:p.Y1021C	0.00086	detected	PIK3CA:NM_006218:exon21:c.A3062G:p.Y1021C	0.01264
#72	detected	PIK3CA gain		undetected		
#73	detected	150: S100A gain; TP53:NM_000546:exon4:c.G355A:p.A119T	0.00996	undetected		
#74	detected	Her2 gain		undetected		
#75	undetected			undetected		
#76	detected	B2M:NM_004048:exon2:c.T128G:p.L43R	0.01661	detected	B2M:NM_004048:exon2:c.T128G:p.L43R	0.03682
#77	detected	CDH1:NM_004360:exon5:c.687+2T>C	0.00770	undetected		
#78	undetected			detected	PIK3CA:NM_006218:exon10:c.A1637G:p.Q546R	0.01684
#79	detected	TP53: NM_000546:exon6:c.672+1G>A	0.03140	undetected		
#80	detected	CDH1:NM_004360:exon2:c.49-2A>G	0.03003	undetected		

#81	undetected			undetected		
#82	undetected			detected	TP53:NM_000546:exon9:c.T979A:p.Y327N	0.05713
#83	detected	PIK3CA:NM_006218:exon21:c.3140A>G, p.H1047R; TP53:NM_000546: c.589G>A, p.V197M	0.0988; 0.1345	undetected		
#84	undetected			undetected		
#85	detected	PIK3CA loss		undetected		
#86	undetected			detected	CDH1:NM_004360:exon8:c.1137+2T>C	0.00792
#87	undetected			undetected		
#88	undetected			undetected		
#89	detected	CDH1:NM_001317184:exon2:c.A77G:p.E26G	0.00708	detected	CDH1:NM_001317184:exon2:c.A77G:p.E26G	0.01174
#90	undetected			detected	TP53:NM_000546:exon11:c.1146delA:p.K382Nfs	0.014175474
#91	detected	TP53:NM_000546: c.724T>C, p.C242R	0.18520	detected	TP53:NM_000546: c.724T>C, p.C242R	0.00548
#92	undetected			undetected		
#93	undetected			undetected		
#94	undetected			detected	CDH1:NM_004360:exon16: c.A2446G:p.K816E	0.01473
#95	detected	CDH1:NM_004360:exon8:c.C1051T:p.Q351X	0.00572	undetected		

NAT: neoadjuvant chemotherapy

Supplementary Table S3. Prognostic impact of adjuvant chemotherapy in the overall cohort and in each subtype of breast cancer

variables	univariate			
	HR	lower	upper	P value
Overall cohort				
No	1			
Yes	1.141	0.601	2.169	0.686
ER(+)Her2(-)				
No	1			
Yes	1.131	0.468	2.733	0.784
ER(±)Her2(+)				
No	1			
Yes*	-	-	-	0.455
TNBC				
No	1			
Yes	1.308	0.426	4.010	0.639

*Three Her2(+) patients received adjuvant chemotherapy, and none had recurrence.

Supplementary Table S4. Patient and tumor characteristics according to the post-NAT ctDNA status

Characteristics	ctDNA not detected (n=64)	ctDNA detected (n=31)	*p* value
Age (mean ± SD)	50.5 ± 9.6	48.9 ± 6.8	0.417
T classification (pre)			0.339
T1	3	0	
T2	34	20	
T3-4	27	11	
N classification (pre)			0.327
N-negative	10	3	
N-positive	54	28	
T classification (post)			0.142
no tumor	11	2	
T1	23	9	
T2	15	14	
T3-4	15	6	
N classification (post)			0.431
N0	21	13	
N1	23	6	
N2	14	8	
N3	6	4	
Response			
pCR	11	2	
non-pCR	52	29	
Molecular type			0.525
ER/PR(+)Her2(-)	30	11	
ER/PR(±)Her2(+)	19	10	
TNBC	15	10	
Chemotherapy			
Anthracycline	53	24	0.591
Taxane	53	27	0.529
Trastuzumab/pertuzumab	19	10	0.799

Supplementary Table S5. Multivariate analysis of recurrence-free survival among patients with detectable ctDNA (n=72)

	multivariate			
Variables	HR	lower	upper	P value
T classification (post)				
no tumor	1			
T1	0.357	0.064	1.998	0.241
T2	0.510	0.083	3.123	0.467
T3-4	0.902	0.169	4.799	0.904
N classification (post)				
N0	1			
N1	1.834	0.505	6.665	0.357
N2	1.653	0.631	4.326	0.306
N3	4.526	1.451	14.115	0.009
Response				
pCR	1			
non-pCR	0.902	0.169	4.799	0.904
ctDNA after NAT				
undetected	1			
detected	8.022	3.241	19.860	<0.001

NAT: neoadjuvant chemotherapy

Supplementary Table S6. Univariate and multivariate analysis of recurrence-free survival in patients with Her2(+) breast cancer and TNBC

variables	univariate				multivariate			
	HR	lower	upper	P value	HR	lower	upper	P value
Age (>50 vs. ≤50)								
T classification (before NAT)								
T1-2	1							
T3-4	0.499	0.169	1.474	0.208				
N classification (before NAT)								
N-negative	1							
N-positive	2.176	0.508	9.314	0.295				
T classification (after NAT)								
no tumor	1							
T1	1.590	0.321	7.878	0.570	0.909	0.167	4.952	0.912
T2	6.574	1.414	30.565	0.016	2.461	0.435	13.917	0.308
T3-4	6.032	1.166	31.202	0.032	4.082	0.756	22.038	0.102
N classification (after NAT)								
N0	1							
N1	1.735	0.651	4.627	0.271	1.845	.633	5.378	0.262
N2-3	3.865	1.329	11.239	0.013	3.753	1.146	12.297	0.029
Response								
pCR	1							
non-pCR	3.328	0.777	14.243	0.105	4.082	0.756	22.038	0.102
ctDNA								
before NAT*	1.028	0.445	2.372	0.949				
after NAT*	3.958	1.678	9.336	0.002	3.123	1.139	8.564	0.027
Genes								
TP53#	2.089	0.848	5.145	0.109				
CDH1#	0.233	0.031	1.731	0.154				
PIK3CA#	2.371	0.871	6.455	0.091				

*The presence of ctDNA vs. nonpresence of ctDNA; #gene mutation vs. nonmutation

NAT: neoadjuvant chemotherapy

Supplementary Figure S1

A

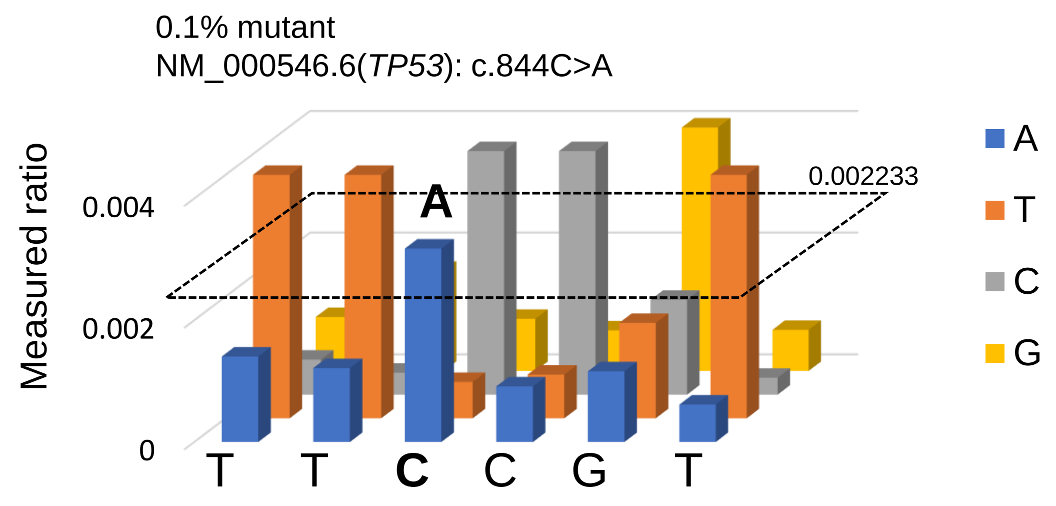

B

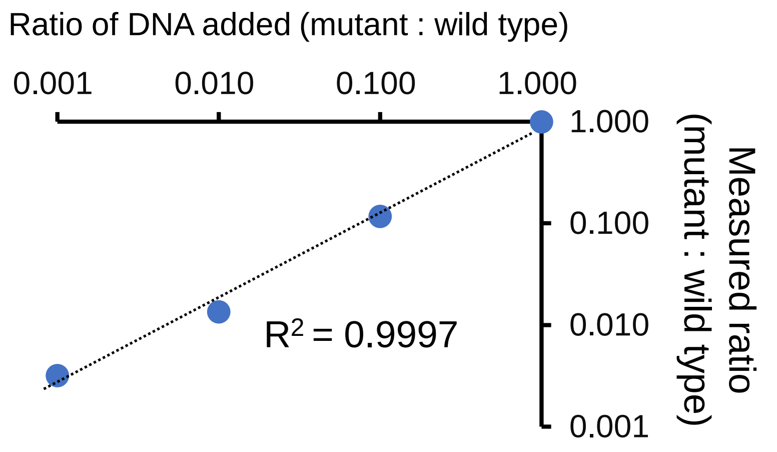

added DNA	measured DNA
100.00%	0.994981
10.00%	0.117648
1.00%	0.013485
0.10%	0.003177

Deep sequencing performance for the reference variant NM_000546.6(TP53): c.844C>A. (A) Each bar indicates the frequency of correct and erroneous nucleotides from sequencing within the region of the reference variant. The mean background error per base was 9.2×10^{-4}, and the upper limit of the 99% confidence interval of the background error was 2.23×10^{-3} (dotted plane). A variant was considered correct when the frequency of variants was more than 2.23×10^{-3}. The measured frequency of the 0.1% reference variant was 3.17×10^{-3}, which was higher than upper limit of background errors, showing that the variant was detected. (B) High degree of linearity between the added and measured ratio of variants.

Supplementary Figure S2

Tumor **Before NAT** **After NAT**

Patient #8 : TP53:NM_000546: c.916C>T, p.Arg306* (NC_000017.10:g.7577022G>A)

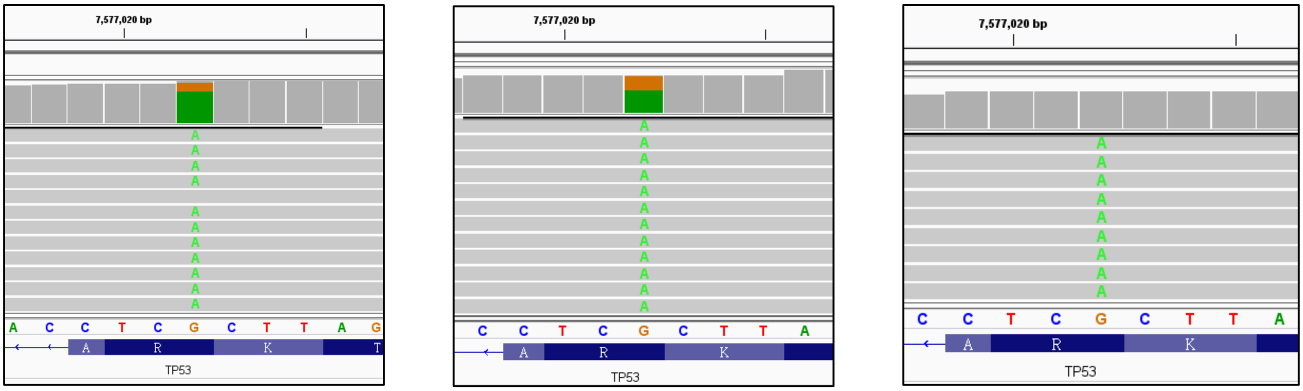

Patient #91: TP53:NM_000546: c.724T>C,p.Cys242Arg (NC_000017.10:g.7577557A>G)

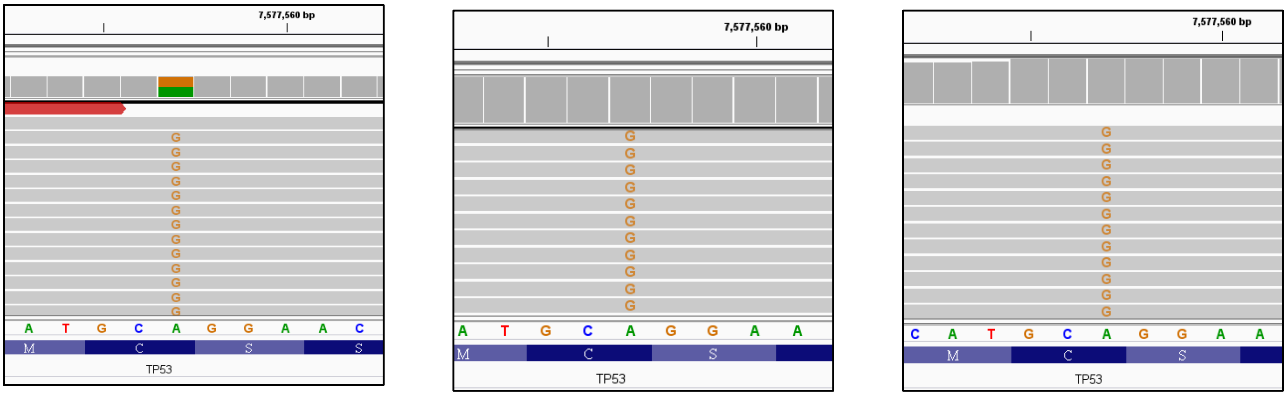

Patient #45: TP53:NM_000546: c.818G>A, p.Arg273His (NC_000017.10:g. g.7577120C>T)

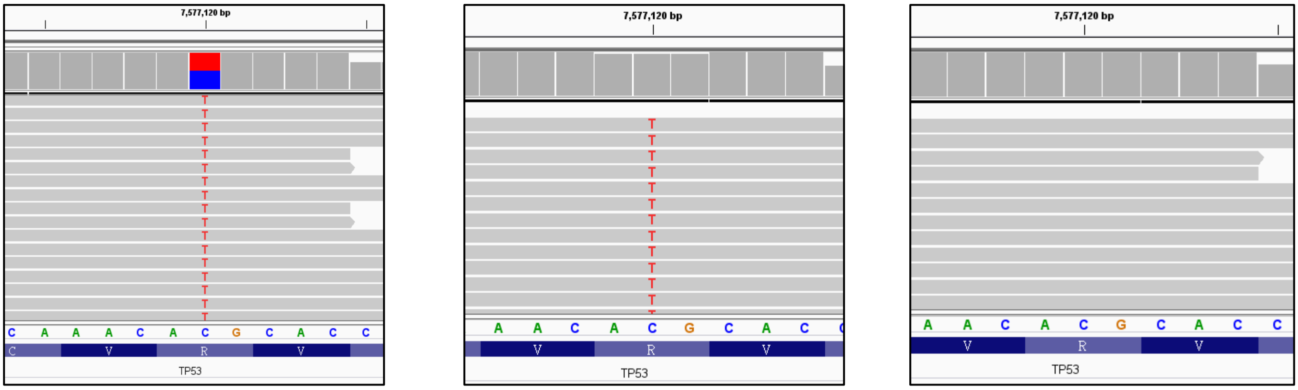

Supplementary Figure S2 (continued)

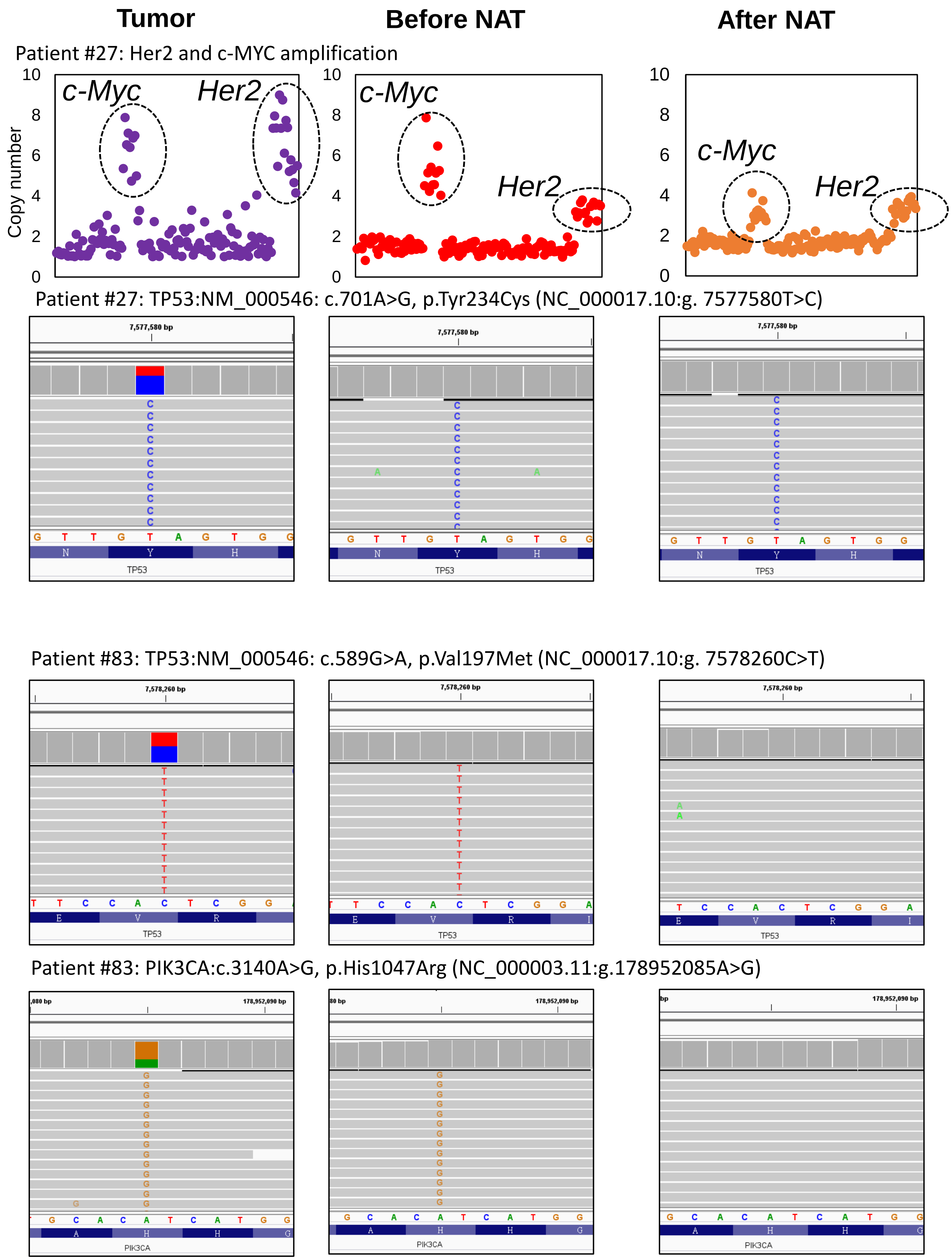

Supplementary Figure S2 (continued)

Illustration of the genetic alterations in DNA of breast tumor, ctDNA before NAT and ctDNA after NAT**.** We selected 5 patients who had *TP53* variants in their ctDNA and their biopsy tumors were available for DNA extraction. We performed NGS on the DNA of the biopsy tumor to check whether the *TP53* variants that were detected in ctDNA existed in the DNA of breast tumors. If the *TP53* variant could be parallel identified in the DNA of the breast tumor and ctDNA, we could confirm that the source of the genetic variants detected in the ctDNA originated from breast tumors.

The *TP53* gene variants of patients #8 and #91 were detected in DNA of breast tumors, ctDNA before and after NAT. These results suggest that the *TP53* variants in the ctDNA came from breast cancer cells. The presence of ctDNA after NAT was related to a high risk of recurrence; accordingly, both patients experienced recurrence.

For patient #45, the source of the *TP53* variant (*TP53*:NM_000546: c.818G>A) was detected both in DNA of breast tumors and the ctDNA before NAT, suggesting the variants of ctDNA originating from breast tumor. The variant was not identified in the ctDNA after NAT. She did not experience recurrence.

Patients #27 and #83 had more than one genetic alteration in their tumors; these mutations have the potential to be biomarkers for the presence of ctDNA. Patient #27 had *Her2* and *c-MYC* amplification in the breast tumor, ctDNA before and after NAT. The tumor DNA also contained the mutation *TP53*:NM_000546: c.701A>G, and this variant also observed in ctDNA before and after NAT. Patient #27 experienced recurrence. Patient #83 had variants in the *TP53* and *PIK3CA* genes in tumor cells. Both variants were identified in ctDNA before NAT but not after NAT. Patient #83 did not experience recurrence. The data show good concordance between *TP53* variants and other genetic alterations in ctDNA.

Supplementary Figure S3

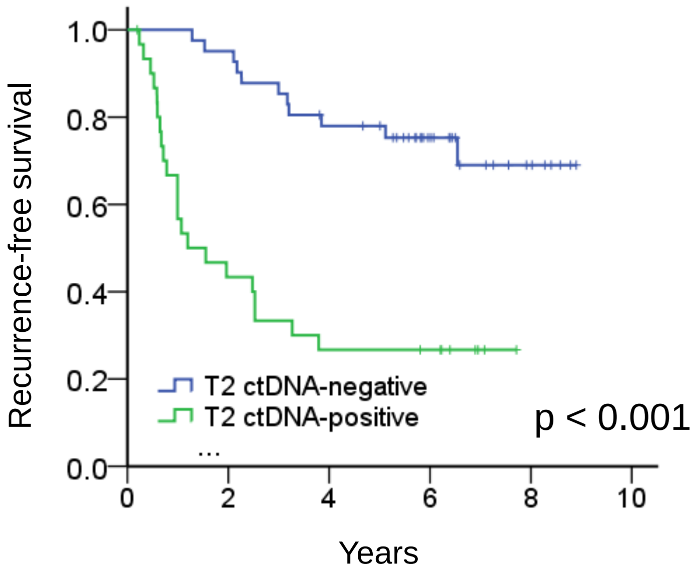

ctDNA positivity after NAT was significantly associated with RFS among 72 patients with detectable genetic variants before and after NAT, $p < 0.001$.

Supplementary Figure S4

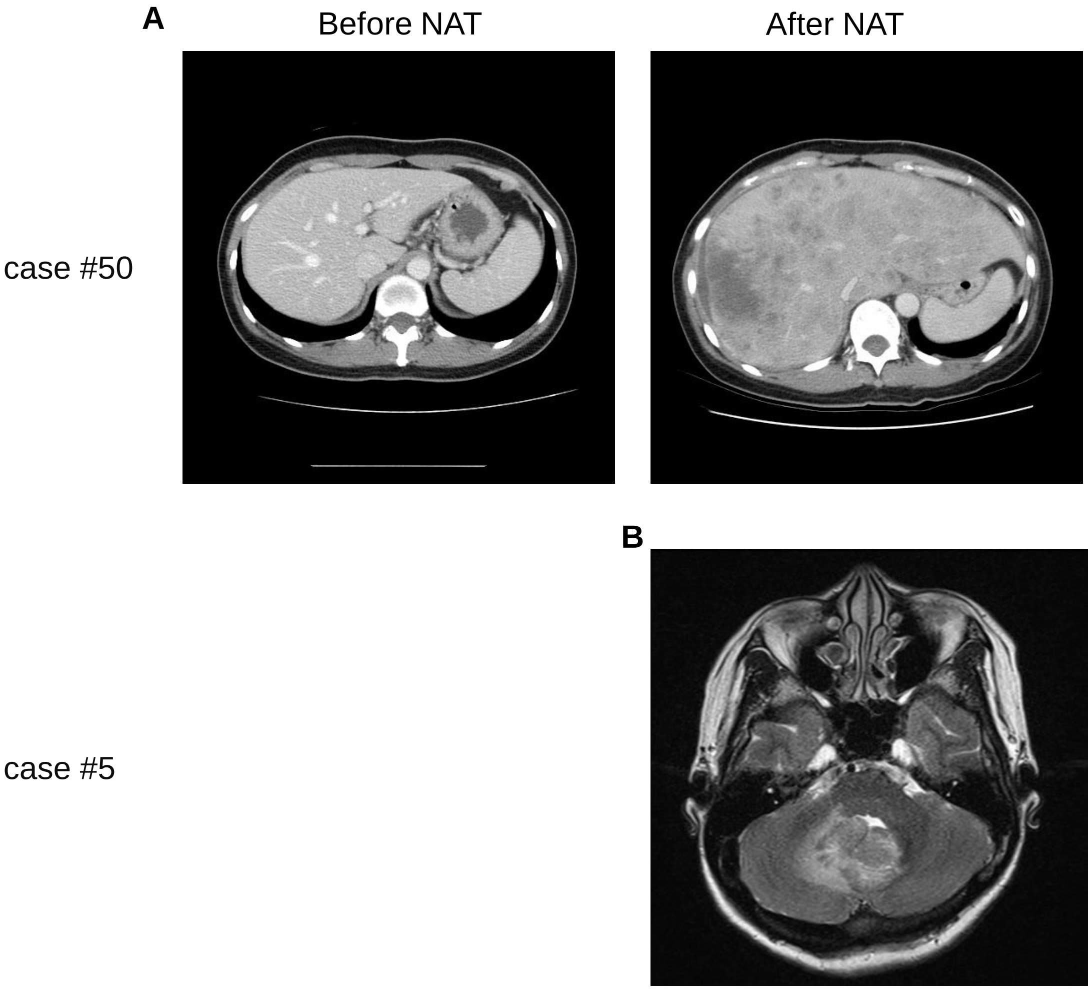

For case #50, staging computed tomography (CT) showed no hepatic metastasis before NAT; however, follow-up CT revealed multiple hepatic metastases 6 months after NAT. Case #5 did not have neurologic symptoms before NAT. However, unsteady gait occurred 13 months after NAT, and magnetic resonance imaging found cerebellar metastasis.
